# Announcement

**DOI:** 10.1177/2041669515599331

**Published:** 2015-07-23

**Authors:** Pete Thompson, Tim Meese, Frans Verstraten, Johan Wagemans

**Affiliations:** Managing Editors

This is the first issue of *Perception* under new ownership. The Pion stable of journals, which includes both *Perception* and *i-Perception*, has been acquired by SAGE publications. This move follows the death of Pion’s founder and owner, Adam Gelbtuch, last year.

*Perception* has always had a personality distinct from other journals in the field, largely due to the influence of Richard Gregory, the founding editor. It retains this distinctive character to this day; a journal with editorials, book reviews, and even cartoons is slightly unusual. Furthermore, *Perception* has never shied away from publishing papers of an eclectic or unusual nature that might not fit the mould of other journals. Above all, *Perception* has always enjoyed extremely high production values; Adam Gelbtuch’s expertize in publishing very high-quality books with color plates is very much in evidence in *Perception*’s appearance.

It is clear that SAGE appreciate the unique nature of their new acquisitions and early discussions with us, the managing editors, have been immensely reassuring; very little has changed in this first issue under their stewardship, as you can see, and the publishers seem committed to preserving both *Perception*’s and *i-Perception*’s unique character in the future. Moreover, happily, SAGE intends to make no changes to the Editorial Board and Gillian Porter will continue as our Administrative Manager.

There will be changes of course, for example, manuscript submission will be transitioning to ScholarOne Manuscripts in the coming weeks, but we look forward to *Perception*—and *i-Perception*—being part of a large, independent publishing company with a first-class reputation in journal publishing.

